# Unique properties of PTEN-L contribute to neuroprotection in response to ischemic-like stress

**DOI:** 10.1038/s41598-019-39438-1

**Published:** 2019-02-28

**Authors:** Magdalena C. E. Jochner, Junfeng An, Gisela Lättig-Tünnemann, Marieluise Kirchner, Alina Dagane, Gunnar Dittmar, Ulrich Dirnagl, Britta J. Eickholt, Christoph Harms

**Affiliations:** 1 Charité–Universitätsmedizin Berlin, corporate member of Freie Universität Berlin, Humboldt Universität zu Berlin, and Berlin Institute of Health, Neurocure Cluster of Excellence, Department of Experimental Neurology, Berlin, Germany; 2 Center for Stroke Research Berlin, Charité–Universitätsmedizin Berlin, Berlin, Germany; 3 Medical Research Centre, The First Affiliated Hospital of Zhengzhou University, Zhengzhou, China; 4 Max Delbrück Centre for Molecular Medicine (MDC), Proteomics Platform, Robert-Rössle-Straße 10, 13125 Berlin, Germany; 5 Berlin Institute of Health (BIH), Proteomics Platform, 10178 Berlin, Germany; 6 Berlin Institute of Health (BIH), QUEST–Centre for Transforming Biomedical Research, 10178 Berlin, Germany; 7 German Centre for Neurodegenerative Diseases (DZNE), Berlin, Germany; 8 Charité–Universitätsmedizin Berlin, Institute of Biochemistry, Berlin, Germany; 9 Proteome and Genome Research Laboratory, Luxembourg institute of Health, 1a Rue Thomas Edison, 1224 Strassen, Luxembourg

## Abstract

Phosphatase and tensin homolog (PTEN) signalling might influence neuronal survival after brain ischemia. However, the influence of the less studied longer variant termed PTEN-L (or PTENα) has not been studied to date. Therefore, we examined the translational variant PTEN-L in the context of neuronal survival. We identified PTEN-L by proteomics in murine neuronal cultures and brain lysates and established a novel model to analyse PTEN or PTEN-L variants independently *in vitro* while avoiding overexpression. We found that PTEN-L, unlike PTEN, localises predominantly in the cytosol and translocates to the nucleus 10–20 minutes after glutamate stress. Genomic ablation of PTEN and PTEN-L increased neuronal susceptibility to oxygen-glucose deprivation. This effect was rescued by expression of either PTEN-L indicating that both PTEN isoforms might contribute to a neuroprotective response. However, in direct comparison, PTEN-L replaced neurons were protected against ischemic-like stress compared to neurons expressing PTEN. Neurons expressing strictly nuclear PTEN-L NLS showed increased vulnerability, indicating that nuclear PTEN-L alone is not sufficient in protecting against stress. We identified mutually exclusive binding partners of PTEN-L or PTEN in cytosolic or nuclear fractions, which were regulated after ischemic-like stress. GRB2-associated-binding protein 2, which is known to interact with phosphoinositol-3-kinase, was enriched specifically with PTEN-L in the cytosol in proximity to the plasma membrane and their interaction was lost after glutamate exposure. The present study revealed that PTEN and PTEN-L have distinct functions in response to stress and might be involved in different mechanisms of neuroprotection.

## Introduction

Phosphatase and tensin homolog -long (PTEN-L or PTENα) is a longer variant of the lipid and protein phosphatase PTEN. From an alternative start codon in frame with the PTEN sequence, 173 additional amino acids (aa) are translated N-terminal of PTEN^1^. PTEN acts as a tumour suppressor, antagonising the PI3K/AKT pathway at the plasma membrane, among other functions^2^. Additionally, PTEN has been characterised as present in the nucleus of a number of cells, including fully differentiated neurons. It is thought that nuclear localisation of PTEN is a dynamic process that correlates with cell cycle progression and the cellular differentiation state, which can be triggered by cellular insults such as ischemia^3,4^. To date it is unclear whether nuclear translocation of PTEN is beneficial or detrimental for cellular survival after ischemic-like stress^5–7^. Since previous studies were conducted before PTEN-L was discovered, it is also unknown if PTEN-L contributes to a neuroprotective effect. PTEN-L and its N-terminal 173 aa region are intrinsically disordered^8^. Intrinsically disordered proteins have been described as hot-spots for post-translational modifications and protein-protein interactions^9^. This led us to hypothesise that PTEN-L might modulate signaling after ischemia dependent on distinct protein-protein interactions. To examine our research questions, we developed an *in vitro* model to be able to compare different PTEN variants in primary neurons in the absence of endogenous PTEN. We aimed to investigate the subcellular localisation of both PTEN variants and cellular survival after ischemic-like stress. Furthermore, we analysed the compartment-specific protein interactome of both PTEN variants before and after ischemic-like stress with the goal to identify novel targets of endogenous neuroprotection. In the present study, we used oxygen-glucose deprivation and exposure to 50 µM glutamate to apply ischemic-like stress to neurons *in vitro*. Both methods lead to neurodegeneration and cell death, which is mostly caused by intracellular calcium overload triggered by excess influx of extracellular calcium through NMDA receptor gated channels^10,11^. Our data characterise distinct phenotypes of PTEN and PTEN-L in response to ischemic-like stress: PTEN-L localised predominantly in the cytoplasm and enriched in a complex with Gab2 at the outer cell membrane. In response to glutamate stress, PTEN-L showed rapid dissociation from this complex and partly migrated to the nucleus within 10–20 min, unlike the shorter PTEN variant. Genomic ablation of PTEN and PTEN-L led to increased vulnerability and cell death when neurons were exposed to oxygen-glucose deprivation. Replacement with either PTEN or PTEN-L rescued the susceptible phenotype of PTEN loss. However, in direct comparison we observed a significant survival benefit of neurons expressing the PTEN-L variant. In addition, PTEN and PTEN-L had a distinct protein interactome in response to ischemic-like stress, which indicates that the intrinsically disordered N-terminal extension of PTEN-L might incorporate unique regulatory properties.

## Results and Discussion

### PTEN-L is expressed in the brain and in primary neuronal cultures

**Figure 1:**
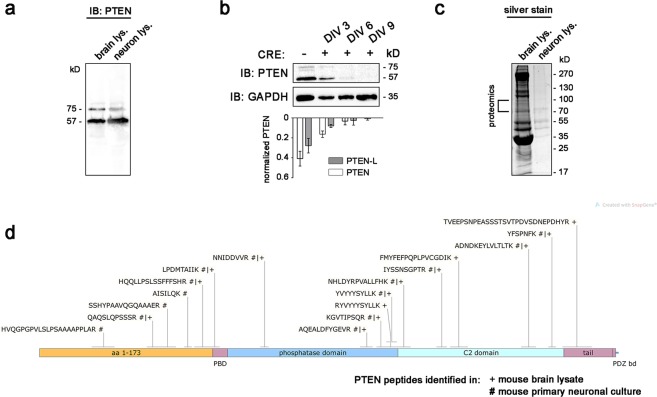
PTEN-L is expressed in the mouse brain and in primary neuronal cell cultures. (**a**) Bands higher than 57 kD were observed, when primary cortical culture lysate or adult brain lysate derived from 8 week old C57BL/6 mice were blotted against PTEN. (**b**) Primary neuronal cultures from conditional PTEN knockout mice were transduced with Cre delivering lentiviral particles. Immunoblotting against PTEN at different time points after Cre transduction showed that PTEN protein isoforms around 57 kD and higher bands around 70–75 kD gradually decreased between day 6 and day 9 in culture, indicating genomic ablation of PTEN and PTEN-L (quantification based on n = 3 independent experiments. Values normalised against total PTEN intensity on each blot). Uncropped immunoblots are available under 10.6084/m9.figshare.7472984. (**c**) To confirm the identity of PTEN positive bands with a higher molecular weight than 57 kD, PTEN was purified using HiTrap heparin columns and subsequent immunoprecipitation from primary cortical cultures or adult cortex derived from 8 week old C57BL/6 mice. Gel pieces were extracted from silver stained gels for proteomics analysis. 500 µg of total protein was used as an input for each purification and 20% of the eluated protein fraction was loaded on the gel. (**d**) Six peptides were identified in mouse cortical neurons # and three peptides in adult brain lysate + that matched to the amino acid (aa) sequence unique to PTEN-L (aa 1–173) by LC-ESI-MS analysis.

First, we identified endogenous PTEN-L in mouse whole brain lysates and neuronal cultures. Immunoblotting with a C-terminal PTEN antibody detected consistently the presence of two major PTEN bands (Fig. 1a). One band exhibited the apparent molecular weight of the 403 aa PTEN protein (57 kilo Dalton (kD)), the other had a molecular weight of 75 kD, which closely resembles the predicted weight of the PTEN-L variant^1,12^. In order to unequivocally characterise the upper band as PTEN variant, we tested if it was sensitive to genetic knockout of PTEN in neurons derived from conditional PTEN knockout mice. In response to *Cre* delivery via transduction with lentiviral particles (LVPs), both the 57 kD band and bands of higher molecular weight (~70–75 kD) started to fade at day *in vitro* three (DIV 3) and disappeared at DIV 9 (Fig. 1b), indicating that the upper band detected by the PTEN antibody truly represents a PTEN variant. We confirmed the identity of the upper bands extracted from silver stained gels by mass spectrometry (Fig. 1c): Six peptides in neuronal culture samples and three peptides in whole brain samples of adult mice matched the amino acid sequence unique to PTEN-L (aa 1–173) (Fig. 1d). Additional matches to the sequence shared by PTEN and PTEN-L (aa 174–576) were identified. A second run with samples from an independent experiment confirmed the identity of four peptides in the PTEN-L sequence (Supplementary Fig. 1). Taken together, our experiments indicate that PTEN-L exists in the mouse brain and in mouse primary neurons. Furthermore, a knockout of both PTEN and PTEN-L in primary neurons derived from conditional PTEN knockout mice could be achieved by *in vitro Cre* gene delivery.

### Validation of a PTEN knockout and replacement model in neurons

**Figure 2:**
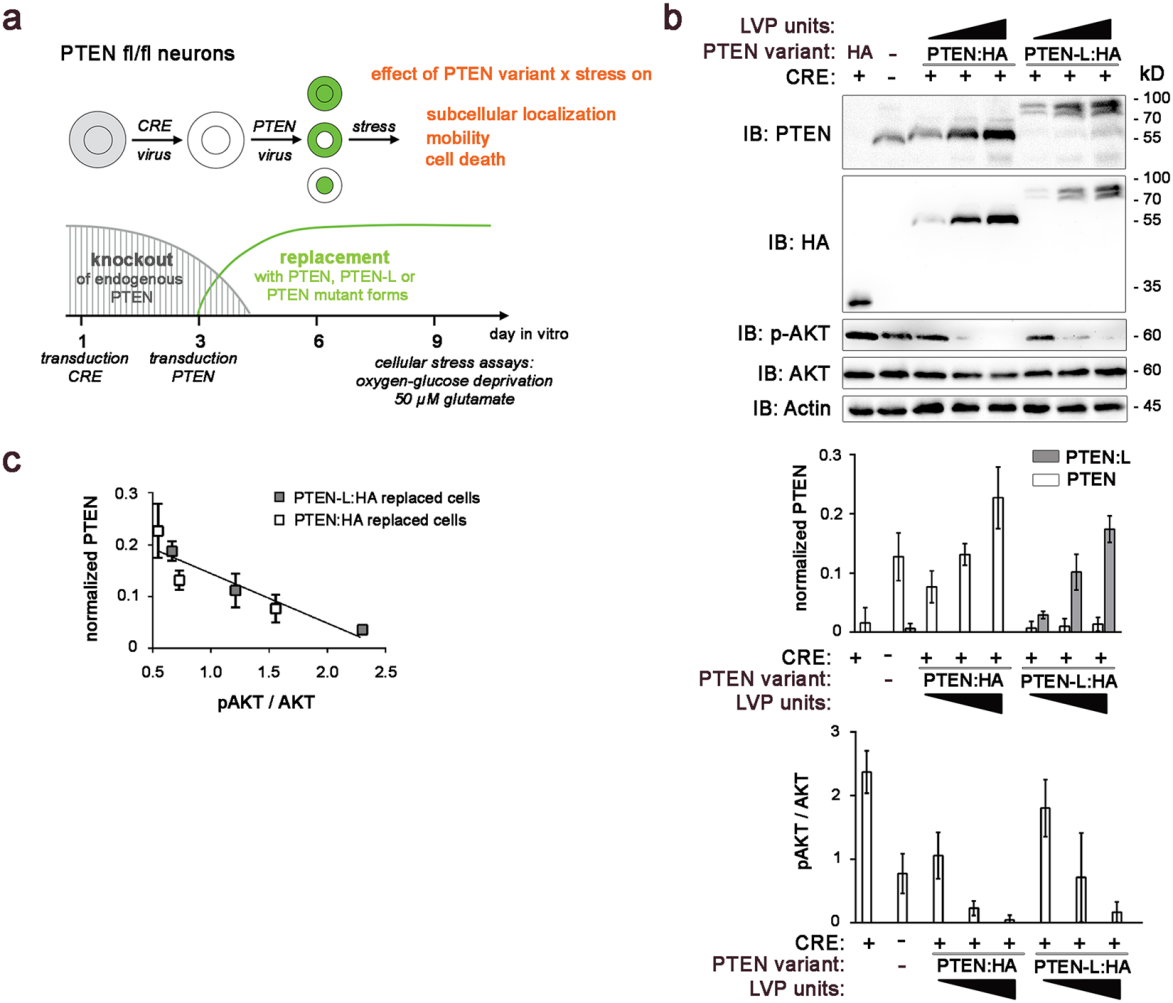
PTEN knockout and replacement model in primary neurons to compare PTEN variants under physiological conditions. (**a**) A replacement model was established to examine the effects of different PTEN isoforms in neurons lacking endogenous PTEN. Neurons derived from conditional PTEN knockout mice were transduced with CRE delivering lentiviral particles (LVPs) to knock endogenous PTEN and transduced with PTEN delivering LVPs to replace with different PTEN isoforms. Various stressors were applied to the PTEN replaced cells to study intracellular shuttling and cell death. (**b**) PTEN isoforms were titrated to replace endogenous total PTEN levels. A dose-dependent reduction of the pAkt/AKT ratio was observed in both cells expressing PTEN:HA and PTEN-L:HA isoforms (quantification based on n = 3 independent experiments. PTEN values normalised against total PTEN intensity on each blot. pAKT values normalised against AKT. Actin served as a loading control). Uncropped immunoblots are available under 10.6084/m9.figshare.7472984. (**c**) A multiple regression analysis was run to predict pAKT/AKT levels from PTEN dose and genotype. These variables significantly predicted pAKT/AKT levels (F(2, 15) = 21.79; p < 0.001. R² = 0.74). PTEN dose added significantly to the prediction (p < 0.001), but PTEN genotype did not (p = 0.364). Data from n = 3 independent experiments is presented as means with standard deviations.

In an effort to study the function of PTEN-L independent of the 57 kD PTEN isoform in neurons and to avoid an overexpression paradigm, we developed a PTEN knockout and replacement model using primary neuronal cultures derived from conditional PTEN knockout mice (Fig. 2a).

After transduction with cre-delivering LVPs at DIV 1 to knock endogenous PTEN variants from conditional PTEN knockout mice, we applied different doses of PTEN-HA or PTEN-L:HA delivering LVPs at DIV 3. Hemagluttinin (HA) tag was fused C-terminally to PTEN or PTEN-L with a preceding spacer. Immunoblots from neuronal lysates harvested at DIV 9 showed a loss of endogenous PTEN expression in cre-transduced neurons and an increase of exogenous PTEN:HA or PTEN-L:HA dependent on viral dose (Fig. 2b). The protein amount of the downstream target pAKT was reduced dependent on the amounts of exogenous PTEN:HA or PTEN-L:HA expressed, indicating a fully active catalytic activity of exogenous PTEN-L:HA (Fig. 2b). Quantification of immunoblots from three independent experiments were used to titrate exogenous PTEN:HA and PTEN-L:HA to match the endogenous PTEN level of wildtype neurons and thus avoid an overall increase of PTEN gene product. In neurons treated with the highest virus titres, which were not used in subsequent experiments, additional weak PTEN-positive and HA-negative bands appeared at 40 kD, 57 kD, and 65 kD. A multiple regression analysis was run to predict pAKT/AKT levels from PTEN dose and genotype. These variables significantly predicted pAKT/AKT levels (F(2, 15) = 21.79; p < 0.001. R² = 0.74). PTEN dose added significantly to the prediction (p < 0.001), but PTEN genotype did not (p = 0.364) (Fig. 2c). This indicates that both PTEN-L:HA and PTEN:HA were able to antagonise AKT phosphorylation in a dose-dependent manner when expressed in neurons lacking endogenous PTEN.

### PTEN-L translocates to the nucleus in response to ischemic-like stress

**Figure 3:**
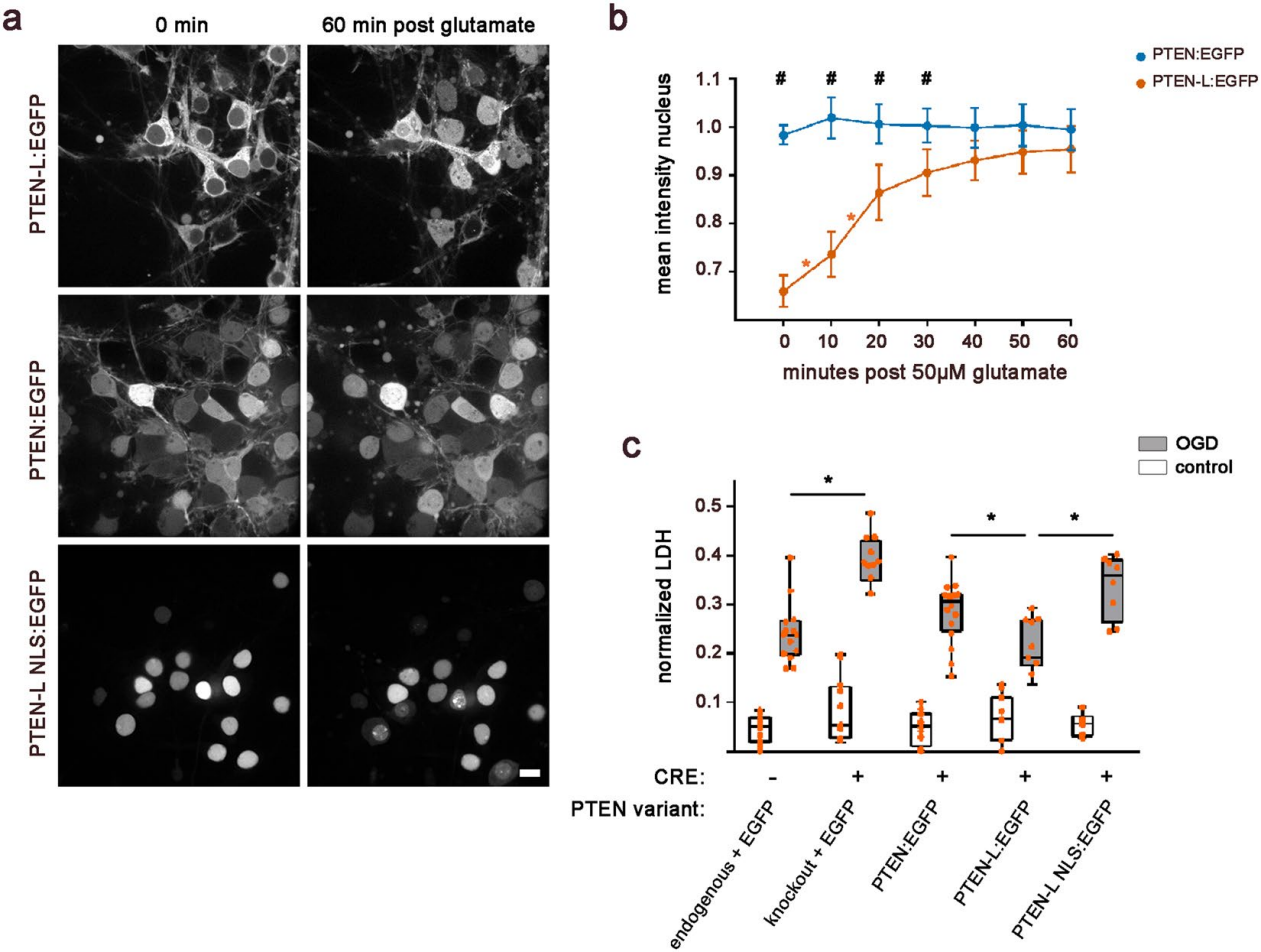
PTEN-L translocates to the nucleus in response to glutamate stress and protects neurons against oxygen-glucose deprivation induced cell death. (**a**) Primary neuronal cultures were replaced with indicated PTEN isoforms and stressed with 50 µM glutamate during live cell imaging. Time lapse movies are available under 10.6084/m9.figshare.7466648. PTEN-L:EGFP localised predominantly in the cytosol and a dynamic shift from the cytosol to the nucleus was observed 60 min after glutamate treatment. PTEN:EGFP was found in both compartments and no change of localisation was observed. As a control, cells were replaced with an immobile nuclear PTEN-L:NLS variant. The scale bar represents 10 µM. (**b**) Nuclear mean intensity was quantified in PTEN-L:EGFP and PTEN:EGFP replaced neurons and normalised by the total intensity of each cell (n = 20–30 cells per group). We analysed if PTEN genotype affected PTEN localisation in response to glutamate stress by two-way ANOVA with repeated measurements. At the time of glutamate treatment, PTEN-L:EGFP showed less signal in the nucleus compared to PTEN:EGFP replaced cells (t(448) = 10.86; p < 0.001). This difference, indicated by #, remained significant when comparing time points 10–20 min, 20–30 min and 30–40 min post glutamate (t(448) = 9,50; p < 0.001; t(448) = 4.77; p < 0.001; t(448) = 3.26; p = 0.005). The increase of PTEN-L:EGFP into the nucleus was steepest between time points 0 min to 10 min (t(384) = 4.21; p < 0.001.) and 10 min to 20 min (t(384) = 7.09; p < 0.001) after glutamate treatment, indicated by *. (**c**) Vulnerability to Oxygen-Glucose deprivation, measured by LDH increase, was compared between cells expressing different PTEN species and parallel control plates by two-way ANOVA. PTEN knockout neurons were more vulnerable than PTEN wildtype neurons (t(110) = 10.34; p < 0.001). Neurons replaced with PTEN:EGFP or PTEN-L:EGFP were rescued compared to PTEN knockout neurons exposed to OGD (t(110) = 7.50; p < 0.01 and t(110) = 11.72; p < 0.001). In direct comparison, PTEN-L replaced neurons showed a survival benefit compared to neurons expressing the shorter PTEN:EGFP variant (t(110) = 3.66; p < 0.001). Neurons expressing immobile PTEN-L NLS:EGFP exclusively in the nuclear compartment fared worse in comparison to PTEN-L:EGFP expressing neurons (t(110) = 7.44; p < 0.001). Data is shown as interquartile ranges with minimum and maximum as error bars. Each dot signifies an independent data point derived from 7 independent experiments.

To study the intracellular dynamics of PTEN-L localisation after stress, EGFP-tagged PTEN constructs were cloned for live cell imaging and titrated as described above to match physiological PTEN protein levels. Enhanced green fluorescent protein (EGFP) was fused C-terminal to PTEN or PTEN-L with a preceding spacer. To generate a PTEN-L construct that localises exclusively in the nucleus of neurons, the nuclear import sequence of T large antigen (Pro-Lys-Lys-Lys-Arg-Lys-Val followed by a Ser-Gly-Gly spacer) was fused N-terminal to PTEN-L. PTEN:EGFP was distributed in the cytosol and nucleus of primary neurons at DIV 9. In contrary, PTEN-L:EGFP predominantly localised in the cytosol largely sparing the nucleus (Fig. 3a). To test the hypothesis that PTEN-L translocates to the nucleus in response to cellular stress and influences cellular survival, we applied 50 µM glutamate and monitored cells by live cell microscopy. The cells expressing PTEN:EGFP, PTEN-L:EGFP, or the nuclear variant PTEN-L NLS:EGFP as a control were imaged every 10 min for 90 min. The time-lapse movies compiled from the 90 min observation, clearly showed a rapid, dynamic translocation of PTEN-L:EGFP from the cytosol to the nucleus in response to glutamate stress (Movie 1), while no shift to the nucleus could be observed in PTEN:EGFP expressing neurons (Movie 2). Neurons with forced nuclear expression of PTEN-L NLS:EGFP are shown as a control (Movie 3). Figure 3a compares the cells at the first time point and 60 minutes after glutamate treatment and an illustration of all frames over 90 minutes is displayed in Supplementary Fig. 2.

Quantification of the observed PTEN-L mobility confirmed that PTEN-L translocated to the nucleus: The normalized mean intensities of EGFP in the nucleus of PTEN-L:EGFP replaced neurons increased in response to glutamate treatment (Fig. 3b). We compared statistically whether PTEN genotype affected PTEN localisation in response to 50 µM glutamate treatment by two-way ANOVA with repeated measures: The mean intensity of the normalised nuclear PTEN signal depended on PTEN genotype and time point after treatment and the interaction between those factors (F(1, 64) = 34.82; p < 0.001. F(6,384) = 40.38; p < 0.001. F(6, 384) = 38.53; p < 0.001). Post-hoc tests confirmed our observation that at the first time point significantly less PTEN-L:EGFP was expressed in the nucleus compared to PTEN:EGFP (t(448) = 10.86; p < 0.001). This difference between nuclear PTEN:EGFP and PTEN-L:EGFP remained significant at time points 10 min, 20 min and 30 min post glutamate treatment (t(448) = 9.50; p < 0.001; t(448) = 4.77; p < 0.001; t(448) = 3.26; p < 0.005) and was not significant at time points 40 min, 50 min and 60 min post glutamate treatment (t(448) = 2.27; p = 0.069; t(448) = 1.87; p = 0.121; t(448) = 1.39; p = 0.167), due to the increase of PTEN-L:EGFP signal in the nucleus. When analysing the dynamic change of the nuclear PTEN-L:EGFP signal over time, the steepest increase was observed between time points 0 min to 10 min (t(384) = 4.21; p < 0.001) and 10 min to 20 min (t(384) = 7.09; p < 0.001)) after glutamate treatment. No significant dynamic increase of nuclear mean intensity was observed in PTEN:EGFP expressing neurons. In summary, experiments revealed that PTEN-L:EGFP was expressed predominantly in the cytosol of primary neurons and dynamically translocated to the nucleus in response to 50 µM glutamate stress. PTEN:EGFP was expressed in both cellular compartments and no translocation was observed by live cell imaging. A previous study showed that nuclear PTEN peaked at 6–9 hours after primary neurons were exposed to NMDA, but no increase was reported at earlier time points after stress^7^. In combination with our results, this suggests that the two PTEN isoforms seem to have a different localisation pattern among cellular compartments of primary neurons and a different dynamic after exposure to ischemic-like stress. The mechanisms of cytosolic – nuclear translocation of PTEN have been examined in other cell types, suggesting that post-translational modifications such as ubiquitination^13,14^, SUMOylation^15^, phosphorylation^16^ or protein-protein interactions such as major-vault protein^17^ might facilitate nuclear import. Furthermore, ubiquitination has been implicated in the translocation of the 403 aa PTEN variant in neurons^7^. However, we did not find an electromobility shift in PTEN-L band size in response to glutamate treatment, which would be expected if ubiquitin or SUMO conjugation had occurred: We performed a cell fractionation to separate nuclear and cytosolic fractions of neuronal cultures replaced with PTEN-L:EGFP, after cultures were treated either with PBS or 50 µM glutamate for 60 min. We harvested the cells either with buffers containing or lacking an inhibitor of de-ubiquitination and de-SUMOylation, N-Ethylmaleimide (NEM). No shift in molecular weight of the PTEN-L and EGFP-positive bands were observed in the cytosolic or nuclear PTEN-L fragments in response to glutamate stress (Supplementary Fig. 3). Furthermore, just one PTEN-L and EGFP-positive band appeared when using buffers with added NEM, which prevented de-ubiquitinating and de-SUMOylating proteases to change the posttranslational modifications of PTEN-L during harvesting. This indicates that the previously observed PTEN-L double band (Supplementary Fig. 3 and Fig. 2b) might be an effect of the harvesting procedure rather than hinting towards a physiologically relevant posttranslational modification. Taken together, the absence of posttranslational modifications suggests a retention of PTEN-L:EGFP to the plasma membrane by other cellular constituents, such as interacting proteins.

### PTEN-L:EGFP improves neuronal survival after ischemic-like stress

Some studies have shown that PTEN translocation is a protective mechanism after ischemic-like stress^6^, while other studies found that preventing translocation is neuroprotective^7^. However, above studies did not differentiate between the different PTEN variants, which may contribute in different ways to a neuroprotective effect. Therefore, we used our model to specifically test how neurons expressing either PTEN or PTEN-L at physiological levels respond to ischemic-like stress. Furthermore, we aimed to investigate whether the presence of PTEN-L in the nuclear compartment is necessary for an endogenous stress response in neurons.

Vulnerability to oxygen-glucose deprivation (OGD) was compared between neurons expressing fully mobile PTEN-L:EGFP and neurons with forced nuclear expression of PTEN-L NLS:EGFP, as well as PTEN:EGFP, PTEN knockout and wildtype neurons. Increase of lactate dehydrogenase (LDH) 24 hours after OGD, indicating cell death, was compared between cells expressing different PTEN species and parallel control plates by two-way ANOVA (Fig. 3c). LDH increase depended on OGD treatment, which explained 70.37% of variance, PTEN genotype, which explained 7.15% and the interaction which explained 4.35% of the total variance (F(1, 110) = 571.00; p < 0.001. F(4,110) = 14.49; p < 0.001. F(4, 110) = 8.82; p < 0.001). Post-hoc tests were calculated to compare the vulnerability of neurons expressing different PTEN variants towards OGD: PTEN knockout neurons were more vulnerable than PTEN wildtype neurons (1.61-fold larger LDH increase; t(110) = 10.34; p < 0.001). Both PTEN:EGFP and PTEN-L:EGFP replaced neurons were rescued compared to PTEN knockout neurons: They showed decreased vulnerability with LDH levels of PTEN:EGFP replaced neurons reduced 1.36-fold (t(110) = 7.50; p < 0.001) and PTEN-L:EGFP replaced neurons 1.84-fold (t(110) = 11.72; p < 0.001). However, neurons replaced with PTEN-L:EGFP showed a significant survival benefit over neurons replaced with the shorter PTEN:EGFP variant with 1.35-fold decreased LDH levels (t(110) = 5.181; p = 0.004). To examine if the presence of PTEN-L in the nuclear compartment was able to protect neurons against ischemic-like stress, we compared neurons replaced with PTEN-L NLS:EGFP to neurons replaced with the mobile PTEN-L:EGFP version: Nuclear PTEN-L NLS:EGFP replaced neurons were as vulnerable towards OGD as PTEN knockout neurons t(110) = 5.15; p = 0.178) and were significantly more vulnerable than neurons expressing mobile PTEN-L:EGFP with a 1.59-fold increased LDH level (t(110) = 7.44; p < 0.001). This indicates that the presence of PTEN-L in the nuclear compartment was not sufficient to protect neurons against ischemic-like stress and that cytosolic PTEN-L is additionally required to provide neuronal resilience against OGD. Representative images of neurons before and after OGD are displayed in Supplementary Fig. 4.

Taken together, we found that PTEN-L:EGFP replaced neurons were able to sustain ischemic-like stress better than neurons expressing immobile PTEN-L in the nucleus, indicating that stress-induced PTEN-L cytoplasmic redistribution or nuclear translocation could have a beneficial effect on neuronal survival. Furthermore, neurons expressing exclusively PTEN-L fared better than neurons expressing the shorter PTEN variant, indicating that PTEN-L might play an important role in the endogenous stress response of neurons. Recent studies examining the function of PTEN-L in other cell types found that PTEN-L might block mitophagy^18,19^ and might improve mitochondrial function by improving ATP production^20^. Our result that neurons expressing PTEN-L tolerated OGD better, might be explained by cytoplasmic redistribution to mitochondria and stabilisation of energy metabolism after OGD. This hypothesis might be supported by our data showing that forced expression of PTEN-L in the nuclear compartment was not protective. Both compartmentalised functions of PTEN-L (cytoplasm containing the mitochondria versus nuclei) might be relevant for the mechanisms of its neuroprotective action. This novel role of PTEN-L in response to stress is surprising and has not been explored in previous studies in the context of brain ischemia. PTEN knockout neurons, on the other hand, showed increased cell death after OGD in our model. As already reported in previous studies^21^, we observed that PTEN knockout neurons had hypertrophic cell somas and an increased neuronal network density compared to wildtype or PTEN replaced neurons (Supplementary Fig. 5). Furthermore, AKT phosphorylation was increased (Fig. 2b and Supplementary Fig. 5). The aberrant morphology of PTEN knockout neurons might have contributed to the increased susceptibility to ischemic-like stress. This is supported by recent studies, which showed that fine-tuned PTEN signalling is important for controlled cell death during neuronal circuit development^22^ and a balanced metabolic state^20^. A balance of PTEN signalling seems to be highly relevant for neuronal survival and resilience.

### The nuclear and cytosolic interactome of PTEN-L and PTEN differs largely and changes in response to glutamate stress

**Figure 4:**
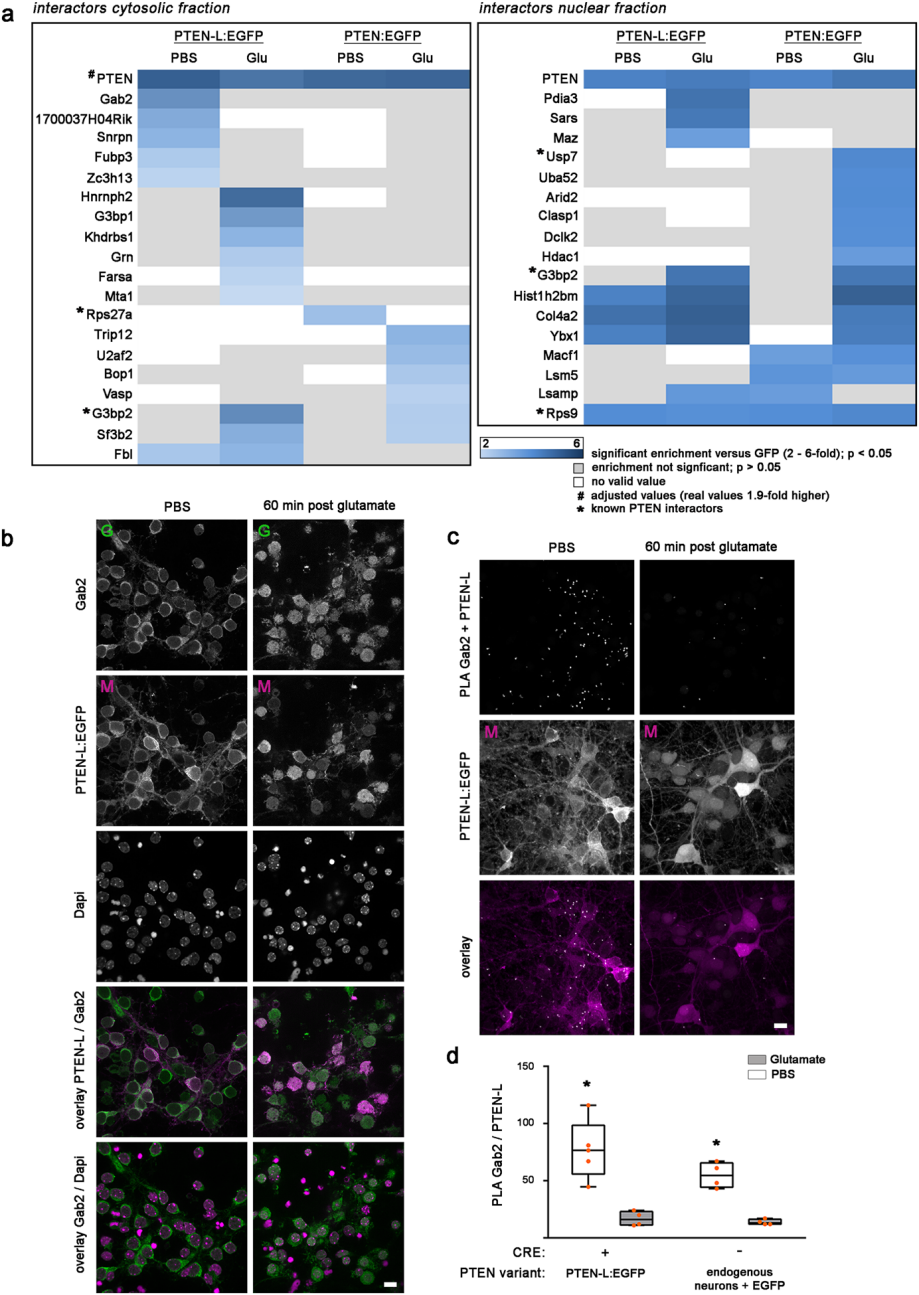
Unique interactome of PTEN-L:EGFP in response to glutamate stress and cytosolic interaction with Gab2. **(a)** A cell fractionation and a pull-down via EGFP was performed in neurons replaced with PTEN or PTEN-L and treated for 60 min with 50 µM glutamate or PBS as a control (n = 4 independent experiments). Interactors of PTEN and PTEN-L in each condition were identified by mass spectrometry: Interactors which were significantly enriched 2–6-fold compared to an EGFP control (p < 0.05) are displayed in blue and were sorted according to enrichment strength. Unique interactors, which were enriched exclusively in one treatment condition and genotype, are displayed first and are followed by common or not glutamate-regulated interactors. PTEN, which is shown as a control, was highly enriched in all fractions and values were adjusted in the cytosolic panel for better visibility (Enrichment of PTEN was 1.9-fold higher than displayed). Previously identified PTEN interactors are marked with a star. Proteins, which did not significantly enrich compared to EGFP are shown in grey and white indicates that no valid value was gathered. (**b**) PTEN-L:EGFP replaced neurons were fixed 60 min post glutamate or PBS treatment and tested for Gab2 expression by immunocytochemistry. Gab2 localised in the cytosol in close proximity to the plasma membrane under PBS conditions and was found in both the nuclear and the cytosolic compartment 60 min after glutamate stress. (**c**) A proximity-ligation assay with a Gab2 and PTEN-L antibody was performed in PTEN-L expressing neurons. We observed an interaction between Gab2 and either exogenous or endogenous PTEN-L under PBS conditions, which was strongly reduced 60 min after 50 µM glutamate was applied. All images quantified are available under 10.6084/m9.figshare.7473332. (**d**) Quantification of interaction complexes (n = 4–5 pictures/condition) showed a significant reduction of interaction complexes after glutamate exposure compared to PBS treatment in neurons expressing exogenous PTEN-L:EGFP (t(7) = 4.50; p = 0.003) and wildtype neurons (t(6) = 7.19; p < 0.001).

To further characterise the observed translocation mechanism of PTEN-L and to compare the contribution of each PTEN isoform to an endogenous stress response, we chose an unbiased proteomics approach to identify interacting proteins. Neurons were replaced with either PTEN-L:EGFP or PTEN:EGFP and harvested 60 min after 50 µM glutamate or PBS control treatment. We then performed a cell fractionation to separate the nuclei from the cytosolic compartments and a pull-down of PTEN variants via GFP-Trap. EGFP expressing wildtype neurons served as a control for unspecific enrichment of interacting proteins via GFP-Trap. Interaction was defined by significant enrichment in comparison to EGFP (Fig. 4a and Supplementary Tables 1,2). This protocol identified 14 proteins in the nuclear and cytosolic fractions, which were significantly enriched by immunoprecipitation with PTEN-L:EGFP, but not with PTEN:EGFP. Five of those proteins enriched with PTEN-L:EGFP in the cytosolic compartment when neurons were treated with PBS and not glutamate (Gab2, 1700037H04Rik, Snrpn, Fubp3 and Zc3h13). GRB2-associated-binding protein 2 (Gab2) was highly enriched with cytosolic PTEN-L:EGFP (3.68 log 2 fold enriched versus GFP control) and showed a strong, 4-fold reduction in binding after glutamate treatment (1.39 log 2 fold enriched versus GFP control). Another group of 6 proteins were precipitated by PTEN-L:EGFP in the cytosolic compartment when neurons were exposed to glutamate stress, but not under PBS conditions (Hnrnph2, G3bp1, Khdrbs1, Gm, Farsa, Mta1). In the nuclear compartment, the proteins Pdia3, Sars and Maz were enriched with PTEN-L:EGFP when neurons were exposed to glutamate stress (Fig. 4a and Supplementary Table 2). A number of proteins interacted with both PTEN isoforms in the nuclear compartment (see Fig. 4a and Supplementary Tables 1,2 for complete list of interactors). Surprisingly, PTEN-L and PTEN had a largely different stress-induced interactome and overlapping stress regulated interactions were sparse: Only G3bp2 was induced in both compartments and both genotypes upon glutamate treatment. PTEN-L and its N-terminal intrinsically disordered region might provide highly specific protein-protein interactions scaffolds^8,23^. Those type of interactions have been described as potentially highly specific but with low affinity permitting rapid binding and release of interacting proteins^9^. This might explain that PTEN-L has a unique interactome which is rapidly regulated in response to ischemic-like stress.

Among the large number of novel interacting proteins identified in the present study, we chose to further characterise the PTEN-L and Gab2 interaction. Gab2 was the most highly enriched protein in the cytosolic fraction and is known to localise at the cell membrane^24^ and interact with the p85 regulatory subunit of PI3K^25,26^.

### Interaction between cell membrane-bound Gab2 and PTEN-L is lost after glutamate stress

To verify Gab2 interaction with PTEN-L and to test if the interaction is stress-specific, we fixed either PBS-treated or glutamate-treated PTEN-L:EGFP replaced neurons after 60 min of treatment. Immunocytochemistry with an antibody against Gab2 revealed a specific localisation of Gab2 at the membrane and a pronounced change in localisation when neurons were exposed to glutamate stress (Fig. 4b). We verified the specificity of the anti-PTENα antibody to detect PTEN-L (Supplementary Fig. 6) and performed a proximity-ligation assay (PLA). Fluorescent PLA-complexes indicative of proximities of the secondary antibodies lower than 40 nm were detected in PBS-treated neurons, indicating direct interaction between PTEN-L and Gab2. Interaction appeared to be strongly reduced in neurons treated with 50 µM glutamate for 60 min (Fig. 4c). Quantification of interaction complexes of n = 4–5 wells/condition revealed a significant decrease of interaction under glutamate treatment (4.61-fold decrease in glutamate compared to PBS condition t(7) = 4.50; p = 0.003). Total cell number was similar in PBS and glutamate treated cells (PBS: 14.8 cells/picture; Glutamate: 15.75 cells/picture). We repeated the PLA with wildtype neurons expressing endogenous PTEN variants and again found a strong interaction between PTEN-L and Gab2 under PBS, but not glutamate conditions (3.98-fold decrease in glutamate compared to PBS condition t(6) = 7.19; p < 0.001) (Fig. 4d).

In line with our proteomics results, we found that Gab2 interacted with PTEN-L in proximity to the cell membrane in the cytosol of neurons. When neurons were exposed to glutamate stress and PTEN-L translocated to the nucleus, the interaction with Gab2 was lost. This was paralleled by a redistribution of Gab2 that showed less membrane-bound Gab2 and localisation of Gab2 in both cytosolic and nuclear compartments of neurons. Similar histological patterns of Gab2 protein in rat and human cortex and hippocampus with a predominant cytoplasmic enrichment at the plasma membrane were observed recently^24^. The study, which examined the effect of Gab2 loss in the brain, found that reduction of Gab2 expression was associated with seizures in patients with temporal lobe epilepsy and in a respective rat model of epilepsy^24^. Instead of focussing on overall loss of Gab2, we observed here immediate changes in subcellular distribution 60 minutes after ischemic-like stress (Fig. 4b). Among the few studies examining Gab2 in the brain, Gab2 has been associated with perinatal hypoxic brain injury^27^, the late onset form of Alzheimer’s disease^28,29^ and it plays a role in neuronal branching and differentiation^30,31^. Furthermore, Gab2 was shown to interact with the P85 subunit of PI3K, which is a functional antagonist of PTEN that enables PIP2 phosphorylation and activates the protein kinase AKT^32^. Thus, Gab2 at its central position at the plasma membrane might initiate different intracellular signalling cascades involved in neuronal cell survival and plasticity. The novel interaction of Gab2 with PTEN-L shown here is of particular interest to future studies, since it could regulate the signalling cascades downstream of Gab2, influencing the neuronal fate in different pathological settings.

To conclude, the present data indicates that PTEN-L and PTEN have distinct functions in neurons in the context of brain ischemia. While earlier studies examined PTEN shuttling in response to ischemia or traumatic brain injury as a therapeutic target in general, our results stress that distinguishing the different PTEN isoforms might increase our knowledge of the neuroprotective cascade initiated by an ischemic trigger. We found that in a model with a balanced overall PTEN dose and downstream AKT phosphorylation, PTEN-L localised predominantly in the cytosol with higher levels at the plasma membranes of neurons and translocated to the nucleus within minutes after ischemic-like stress. When tested in an established *in vitro* stroke model, neurons which expressed PTEN-L were protected against ischemic-like stress compared to neurons expressing the shorter 403 aa PTEN variant or nuclear PTEN-L NLS. Thus, nuclear PTEN-L NLS alone was not able to protect neurons against ischemic-like stress and re-distribution of PTEN-L early after an ischemic trigger might be a specific neuroprotective response. We identified novel protein-protein interactions of PTEN-L and PTEN in response to ischemic-like stress in different cellular compartments. Surprisingly, the two PTEN isoforms had a unique stress-regulated interactome, suggesting that PTEN and PTEN-L are independently regulated after ischemic-like stress in neurons. All identified interacting proteins are potential regulators of neuroprotection or recovery after brain ischemia and would merit further investigations in future studies. Of particular interest is the strong interaction between PTEN-L and Gab2 at the plasma membrane, which was lost after glutamate exposure. Since Gab2 is located at a central position at the plasma membrane as an adaptor protein of tyrosine-kinase receptors, its interaction with PTEN-L and stress-induced release of both proteins from the membrane could be part of a rapid coordinated action to withstand neuronal stress.

## Materials and Methods

### Ethical regulations

All data sets were carried out in accordance with relevant guidelines and regulations at Charité–Universitätsmedizin Berlin. Animal experiments on live vertebrates were not performed. Mice were sacrificed under deep anesthesia for preparation of primary neurons under licence number T0046/07 and work with lentiviral particles was approved under licence number 441/06, both to Dr. Christoph Harms (governmental institution for licensing: Landesamt für Gesundheit und Soziales, Berlin).

### Primary neuronal cultures

Primary cortical neuronal cultures were prepared from C57BL/6 (Fig. 1a,c) or conditional PTEN knockout (Fig. 1b, Figs 2–4, and Supplementary Figs 2–4) mouse embryos at embryonic day E15 as described previously^11^. Primary neurons were cultured at the density 1.35 × 10^5^ cells per 1 cm^2^ in neurobasal medium with B27 supplement (Invitrogen).

### Conditional PTEN knockout mice

Breeding pairs were a kind gift of Lloyd Trotman (Pten^tm2.1Ppp^ MGI:2679886)^33^ and backcrossing was performed on a C57BL/6 background for more than 10 generations.

### Mass spectrometry for identification of PTEN species

500 µg protein derived from adult cortex or neuronal cultures were purified by HiTrap Heparin HP column (GE Healthcare) and incubated with 10 µl PTEN (D4.3) XP rabbit monoclonal antibody conjugated to sepharose beads (Cell Signaling Technology) over night at 4 °C for immunoprecipitation of PTEN. Immunoprecipitates were washed four times with 0.5% Nonidet P-40 buffer and boiled in 50 µl of 1 × SDS sample buffer. 10 µl of these samples were loaded on 10% SDS-polyacrylamide gels and transferred onto polyvinylidene fluoride (PVDF) membrane.

For the tryptic in-gel digestion the samples were washed using an automated setup followed by reduction with 1 mM tris(2-carboxyethyl) phosphine (TCEP) and free sulfhydryl groups carbamidomethylated using 5.5 mM chloroacetamide. Subsequently, the samples were incubated with 2.5 µg sequencing grade trypsin (Promega) for 8 h at room temperature. The reaction was stopped by adding trifluoroacetic acid (TFA) to a final concentration of 1%. For the extraction of the peptides the gel pieces were incubated with a solution containing 80% acetonitrile (ACN) and 0.1% formic acid (FA) for 20 min at room temperature^34^. The extraction solution and the reaction mixture were combined and dried down in a SpeedVac. After resuspending the samples in 2% ACN and 1% TFA, they were purified by C_18_ stage-tips (3 M)^35^. The samples were measured by LC-MS/MS on a Q-Exactive Plus mass spectrometer (Thermo Scientific) connected to a Proxeon nano-LC system (Thermo Scientific) in data-dependent acquisition mode using the top 10 peaks for HCD fragmentation. A volume of 5 µl sample was injected and the peptides eluted on in-house prepared nano-LC column (75 µm × 250 mm, 3 µm Reprosil C_18_, Dr Maisch GmbH) with a 44 min gradient from 4 to 76% ACN and 0.1% FA at flow rates of 0.25 µl/min. MS acquisition was performed at a resolution of 70,000 in the scan range from 300 to 1700 m/z. Dynamic exclusion was set to 20 s and the normalised collision energy was specified to 26, the AGC was set to 10^5^ MS^2^ scans were performed at a resolution of 37000 with a scan range 100 to 1700 m/z.

Data analysis was performed using MaxQuant version 1.5.2.8^36^. The Andromeda-based search was carried out using a mouse Uniprot database (Aug 6th 2014) with a false discovery rate (FDR) of 0.01. Carbamidomethylation was set as a fixed modification while oxidized methionine and acetlyated N-termini were set as variable modifications.

### Lentiviral particle generation and transduction

We used a gene synthesis with ATG start codon replacing the CTG alternative start site to generate the human PTEN-L sequence and cloned it intoan ubiquitin-driven second-generation lentiviral transfer vector (FUGW, Addgene plasmid #14883, a gift from David Baltimore)^37^. All lentiviral plasmids with C-terminal fusion of PTEN to HA-tags were inserted by PCR using BamHI and EcoRI restriction sites. PCR primers for PTEN:HA were forward: 5′-GTAGATGGATCCACCATGACCGCAATTATCAAAGAG-3′ and for PTEN-L:HA forward: 5′-GTAGATGGATCCACCATGGAGC-3′. A common reverse primer was: 5′-GCGATGAATTCCTAGGCATAATCGGGAACGTCG-3′. Hemagluttinin tag (Tyr-Pro-Tyr-Asp-Val-Pro-Asp-Tyr-Ala) was fused C-terminal to PTEN with a preceding spacer (Gly-Gly-Gln). All transfer plasmids with C-terminal fusion to EGFP (PTEN:EGFP, PTEN-L:EGFP, and PTEN-L NLS:EGFP) were inserted by PCR using BamHI and AgeI restriction sites. Primers for PTEN:EGFP, were: forward 5′-GCACAGGATCCACCATGACCGCAATTATCAAAGAG-3′ and reverse: 5′-GCATGTACCGGTCCGCCGGTGCCTCCCACCTTGGTGATCTGAGTGTG-3′ (underlined is the Gly-Gly-Thr-Gly-Gly spacer preceding EGFP) and for PTEN-L:EGFP were forward 5′-GCACAGGATCCACCATGGAGCGTG-3′ and reverse: 5′-GCATGTACCGGTCCGCCGGTGCCTCCCACCTTGGTGATCTG-3′ (underlined is the Gly-Gly-Thr-Gly-Gly spacer preceding EGFP). The forward primer for PTEN-L NLS:EGFP (1804 bp product) was 5′-GCACAGGATCCACCATGGTGCCCAAGAAGAAGAGGAAAGTCTCCGGAGGTATGGAGCGTGGTGGGGAAGC-3′ (underlined is the reduced nuclear import sequence of T large antigen^38^: Pro-Lys-Lys-Lys-Arg-Lys-Val followed by a Ser-Gly-Gly spacer). As a reverse primer 5′-GCATGTACCGGTCCGCCGGTGCCTCCCACCTTGGTGATCTG -3′ (Gly-Gly-Thr-Gly-Gly is the spacer preceding EGFP) was used. As a control plasmid for PTEN:HA and PTEN:EGFP constructs, EGFP was fused to HA-tag and inserted in a FUGW transfer vector using BamHI and EcoRI restriction sites. Forward primer: 5′-GCATGGATCCACCATGGTGAGCAAGGGC-3′ and reverse 5′-GCATGAATTCCTAGGCATAATCGGGAACGTCGTATGGATACTGTCCTCCCTTGTACAGGCTCGTCCATGCC-3′ (underlined is the HA-tag). FUGW expressing codon-optimized iCre:IRES-MYC-mCherry were generated by replacing EGFP in FUGW iCre:IRES-EGFP (a kind gift of Hiroshi Kawabe) with mCherry by unidirectional cloning into AgeI and BsrGI sites. FUGW expressing MYC-mCherry served as a control. All vectors were sequence verified. To produce LVPs human embryonic kidney cells were co-transfected with transfer vectors and second generation lentiviral packaging plasmid psPAX2 (Addgene plasmid #12260) and VSV-G envelope expressing vector pMD2.G (Addgene plasmid #12259; psPAX2 and pMD2.G were a gift from Didier Trono). Conditioned HEK cell medium was harvested 48 and 72 h after transfection, filtered with a 0.45 µm filter (Millex) and LVPs were precipitated with PEG-it virus precipitation solution (System Biosciences) overnight. LVPs were then centrifuged for 1 h at 4000 × g and the viral pellet was taken up in PBS (Thermo Fisher). Aliquots were stored at -80 °C until use. For titration, wt neuronal cultures were transduced on DIV 3 to calculate transduction efficiencies (95% of neurons) and multiplicity of infection based on serial dilutions using EGFP or mCherry fluorescence as a reporter. A second titration step was performed in primary neuronal cultures from conditional PTEN knockout mice: Neuronal cultures were transduced with serial dilutions of PTEN delivering LVPs on DIV 3, lysed on DIV 9 and immunoblotted against PTEN. Protein amounts were quantified by densitometry of PTEN positive bands and compared with PTEN expression in wt neurons. The lentiviral particle dilution leading to similar PTEN expression levels observed in wt neurons was used for subsequent experiments. Primary neurons derived from conditional PTEN knockout mice were transduced on preparation day in suspension with LVPs either delivering *iCre* and *mCherry* to knock PTEN or only *mCherry* as a control. On DIV 3 primary neurons were transduced with *Egfp* or hemagluttinin tagged *Pten*-delivering LVPs or *Egfp*-delivering LVPs as a control.

### Oxygen-Glucose deprivation (OGD)

To expose neuronal cultures to ischemic-like stress at DIV 9, cells were washed three times with glucose-free buffer and incubated in a hypoxic chamber at 0.3% oxygen (O_2_) and 5% carbon dioxide (CO_2_). Cells were exposed to 2.5 h of OGD and were re-oxygenated with conditioned medium afterwards. Parallel control plates were washed the same way than experimental plates, but placed in buffer containing 5% glucose into the incubator for the duration of the experiment. Phase contrast microscopic pictures were taken before and 24 after re-oxygenation using an inverted IX81 microscope (Olympus) with 20x objective to assess cell morphology.

### Lactate dehydrogenase assay

To assess cell death, lactate dehydrogenase (LDH) release was measured in neuronal culture medium before OGD treatment and 24 h after re-oxygenation of the cells. A detailed description of the assay was published in Bio-protocols^39^ alongside the original research article where this protocol was used^40^. In short, 50 µl of medium was collected before OGD, 24 h after re-oxygenation and after cell lysis by triton x-100 (full kill). Samples were incubated with the substrates of LDH, β-nicotinamide adenine dinucleotide (β-NADH) and pyruvate. During the catalytic reaction, decrease of β-NADH was monitored by measuring its absorption at 340 nM in a microplate reader and compared with a standard containing 500 units of LDH. The slope of β-NADH decrease in the samples and standard were used to calculate the LDH units in the samples. LDH values of samples taken before and after OGD were then normalised to the maximum release of LDH measured in the full kill sample.

### Immunoblotting

For immunoblotting, cells were harvested in either 1 x sodium dodecyl sulfate (SDS) buffer or 0.5% Nonidet P-40 buffer containing 1 mM Dithiothreitol (DTT) and 10 mM 4-(2-aminoethyl)benzenesulfonyl fluoride hydrochloride and processed as previously decribed^41,42^. Samples were loaded on 10% SDS-polyacrylamide gels and transferred onto polyvinylidene fluoride (PVDF) membrane. Membrane was blocked in 5% milk, incubated with primary antibodies over night at 4 °C and secondary antibodies (1:2500 dilution) for 1 h at room temperature. An enhanced chemiluminescence system (GE Healthcare) was used to detect immunocomplexes. Anti-Actin (1:1000 dilution), anti-AKT (1:1000 dilution), anti-phospho-AKT (Ser473; 1:1000 dilution) and anti-PTEN (1:1000 dilution) antibodies were purchased from Cell Signaling Technology. Anti-GAPDH antibody was obtained from Millipore. Anti-HA.11 antibody was purchased from Covance. Anti-EGFP antibody was purchased from Santa Cruz Biotechnology. In Fig. 2b, for each of the n = 3 independent experiments (Primary neurons derived from embryos of three different mothers) two gels were run and transferred onto PVDF membranes in parallel (30 µg protein lysate/lane). One membrane was sequentially incubated with primary antibodies against HA, PTEN and Actin as a loading control. The other membrane was incubated with first p-AKT and then AKT primary antibody. Between antibodies PVDF membranes were quenched for 20 min with hydrogen peroxide.

### Densitometry

Fiji software^43^ was used to quantify the intensity signal of immunoblots derived from three independent experiments. For the PTEN signal the intensities of bands at 55–65 kD (PTEN) and 70–100 kD (PTEN-L) were measured separately on each blot. Values were transferred to Excel and the measured intensity of each lane was normalised against the total intensity of PTEN signals on a given blot (PTEN and PTEN-L signals added up across all lanes) to account for the difference in expression levels between experiments. Phosphorylated AKT was normalised against matching AKT values. GAPDH (Fig. 1b) and Actin (Fig. 2b) served as a loading control.

### Live cell microscopy

Primary neurons derived from conditional PTEN knockout mice were transduced with both *Cre*-delivering LVPs or control LVPs and *Pten*-delivering LVPs or control LVPs as described above and seeded in 8-well microscopy dishes (Ibidi). DIV 9 primary neurons were imaged while maintaining culturing conditions (20% oxygen, 5% CO_2_ and 37 °C). A confocal microscope (Nikon Ti2) with uniform spinning disk illumination (Andor Borealis), an EMCCD Camera (iXon3 DU-888 Ultra) and 60x Plan Apo oil objective (Nikon) was used to acquire images. 8 positions across 4 wells were selected and images of z-planes (30–40 µM with 1 µM intervals) were taken with a laser exciting at 488 nm (>8 mW; 20%) detecting EGFP and a laser exciting at 561 nm (>15 mW; 9%) detecting mCherry. Next, cells were treated with 50 µM glutamate and each position was imaged every 10 min for 90 min using above described settings.

Icy software^44^ and Fiji software^43^ were used to analyse microscopy data. To quantify mean intensity of EGFP in nucleus and cytosol, regions of interest (ROI: 100 pixel) were defined in the nucleus and cytosol of each cell of a selected z-plane and mean intensity was measured across time points 0 min, 10 min, 20 min, 30 min, 40 min, 50 min and 60 min post glutamate treatment. Later time points were excluded from quantification, since morphological changes in response to glutamate stress made the placement of ROIs imprecise. Values were then normalised against the total mean intensity of each cell in cytosol and nucleus to control for different expression levels.

### Cell fractionation and immunoprecipitation of PTEN:EGFP and PTEN-L:EGFP protein complexes

Primary neurons derived from n = 4 conditional PTEN knockout mice were plated and transduced with cre- and PTEN-delivering LVPs as described above. Neurons were transduced with either PTEN:EGFP, PTEN-L:EGFP or EGFP control and treated with either 50 µM glutamate or PBS for 60 min before they were harvested in 80 µl cold cell lysis buffer per well (10 mM hepes, 2 mM MgCl2, 1 mM EDTA, 1 mM EGTA, 10 mM KCl, 10 mM NaF, 0.1 mM Na3Vo4, pH 7.5). Lysates were incubated with 1% NP-40 for 10 min and centrifuged to separate the crude cytosolic fraction. The pellets containing the cell nuclei were washed two times with cell lysis buffer, incubated with nuclear extraction buffer (25 mM hepes, 500 mM NaCl, 10 mM NaF, 10% glycerol, 0.3% NP-40, 5 mM MgCl2) for 30 min, sonificated 4 × 5 seconds and centrifuged to extract the pure nuclear fractions. Crude cytosolic fractions were purified by ultracentrifugation for 60 min at 40.000 rpm. All buffers used contained protease inhibitors (cOmplete, EDTA-free Protease Inhibitor Cocktail, 04693132001, Roche) and 1 mM DTT.

Pull-down of PTEN protein complexes was performed by incubating the purified nuclear and cytosolic fractions with anti-GFP-coupled magnetic agarose beads (GFP-Trap_M, ChromoTek) for 1 h at 4 °C on tumble end-over-end rotator. Beads were washed three times with tris-buffered saline containing 0.05% NP-40, separated with a magnet and shock frozen for mass spectrometry. Whole cell extracts were harvested in 8 mol urea buffer.

### Analyses of PTEN variant interactomes by mass spectrometry

Trap beads from PTEN pull-downs were resuspended in 50 µl urea buffer (6 M urea, 2 M thiourea, 10 mM HEPES, pH 8.0), reduced for 30 min at RT in 12 mM DTT solution, followed by alkylation by 40 mM chloroacetamide for 20 min in the dark at RT. The samples were first digested using 1 µg endopeptidase LysC (Wako, Osaka, Japan) for 4 h. The samples were diluted by adding 100 µl of 50 mM ammonium bicarbonate (pH 8.5), and finally digested with 1 µg trypsin (Promega) for 16 h. The digestion was stopped by acidifying each sample to pH < 2.5 by adding 10% trifluoroacetic acid solution. Peptides were extracted and desalted using StageTip protocol (Rappsilber *et al*., 2003).

To perform LC-MS/MS analyses, peptides were eluted using Buffer B (80% Acetonitrile and 0.1% formic acid) and organic solvent was evaporated using a speedvac (Eppendorf). Peptide samples were resuspended in Buffer A (3% acetonitrile and 0.1% formic acid) and separated on a reversed-phase column (20 cm fritless silica microcolumns with an inner diameter of 75 µm, packed with ReproSil-Pur C_18_-AQ 1.9 µm resin (Dr. Maisch GmbH)) using a 90 min gradient with a 250 nl/min flow rate of increasing Buffer B concentration (from 2% to 60%) on an easy nLC (nanoHPLC) system (Thermo). Peptides were ionized using an electrospray ionization (ESI) source (Thermo) and analyzed on a Thermo Orbitrap Fusion (Q-OT-qIT, Thermo). The mass spectrometer was run in data dependent mode selecting the top 20 most intense ions in the MS full scans, selecting ions from 350 to 2000 *m*/*z*, using 60 K resolution with a 4 × 10^5^ ion count target and 50 ms injection time. Tandem MS was performed by isolation at 0.7 m/z with the quadrupole, HCD fragmentation with normalized collision energy of 32 and resolution of 15 K. The MS^2^ ion count target was set to 5 × 10^4^ with an maximum injection time of 250 ms. Only precursors with charge state 2–7 were sampled for MS^2^. The dynamic exclusion duration was set to 30 s with a 10 ppm tolerance around the selected precursor and its isotopes.

Data were analyzed using MaxQuant software package (v1.5.3.30). The internal Andromeda search engine was used to search MS^2^ spectra against a decoy mouse UniProt database (MOUSE.2017-01) containing forward and reverse sequences. The search included variable modifications of methionine oxidation and N-terminal acetylation, deamidation (N and Q) and fixed modification of carbamidomethyl cysteine. Minimal peptide length was set to seven amino acids and a maximum of 3 missed cleavages was allowed. The FDR (false discovery rate) was set to 1% for peptide and protein identifications. Unique and razor peptides were considered for quantification. Retention times were recalibrated based on the built-in nonlinear time-rescaling algorithm. MS^2^ identifications were transferred between runs with the “Match between runs” option, in which the maximal retention time window was set to 0.7 min. Protein intensities were normalized using the in-built LFQ (label-free quantification) algorithm. The resulting text files were filtered to exclude reverse database hits, potential contaminants, and proteins only identified by site. Statistical data analysis was performed using Perseus software (v1.5.2.4). Technical and biological replicates for each condition were defined as groups and intensity values were filtered for “minimum value of 3″ per group. After log_2_ transformation missing values were imputed with random noise simulating the detection limit of the mass spectrometer. Imputed values are taken from a log normal distribution with 0.25 × the standard deviation of the measured, log-transformed values, down-shifted by 1.8 standard deviations. Differences in protein abundance between EGFP-PTEN variants and EGFP control samples for the different treatments (PBS control and Glutamate) were calculated using two-sample Student’s t test. Proteins passing the significance cut-off (p-value ≤ 0.05, log2 t-test difference >2) were considered specific PTEN binder.

### Immunocytochemistry and proximity-ligation assay

Primary neurons derived from conditional PTEN knockout mice were plated in 8-well microscopy dishes (Ibidi) and transduced with either cre- and PTEN-delivering LVPs as described above (Knockout and exogenous PTEN replacement condition), or mcherry- and EGFP-delivering control LVPs (Endogenous PTEN condition). At DIV 9 cells were treated with either PBS or 50 µM glutamate for 60 min and fixed with 4% formaldehyde. To test the primary antibodies anti-GAB2 (HPA001368, Sigma-Aldrich), derived from murine origin, and anti-PTEN-L (MABS1680, Merck), derived from canine origin, cells were permeabilised with 0.25% triton X-100, blocked with 0.2% normal donkey serum (ab7475, Abcam) and primary antibodies were applied at 1:500 dilutions over-night. Secondary antibodies alexa Fluor 647 donkey anti-rabbit IgG and alexa Fluor 647 donkey anti-mouse IgG (ThermoFisher) were applied at 1:500 dilutions for 1 h at room temperature.

To detect interactions between GAB2 and PTEN-L below a distance of 40 nm, permeabilisation, blocking and primary antibodies were applied like described above. Duolink® PLA PLUS targeting mouse IgGs and Duolink® PLA MINUS secondary antibodies targeting rabbit IgGs were incubated at ambient temperature overnight. Ligation, rolling circle amplification and hybridization with in the far red detectable olgionucleotides were performed according to the manufacturer’s instructions (Duolink ® PLA, Sigma Aldrich).

### Statistical analysis

In Fig. 2c, the influence of intracellular PTEN level on AKT phosphorylation was calculated by multiple regression analysis with the coefficients PTEN level and PTEN genotype, using IBM SPSS software (Version 22).

In Fig. 3c, LDH increase after OGD was compared between neurons expressing different PTEN species by two-way analysis of variance (ANOVA). Main effects of the two-step factor treatment (OGD versus washing control) and five-step factor genotype (PTEN-L:EGFP, PTEN-L NLS:EGFP, PTEN:EGFP, wildtype and knockout) and the interaction of treatment and genotype were calculated. Effect sizes were reported as % of total variation explained by the main effects and the interaction (standard omega squared^45^). In case of a significant ANOVA, Tukey’s multiple comparisons test was calculated to compare the LDH increase between neurons expressing different PTEN species. Alpha error was set to 0.05. P-values of < 0.05 were considered significant. Significant differences in LDH increase between genotypes were reported as a ratio of the means.

In Fig. 3b, translocation of PTEN isoforms in response to glutamate stress was analysed by two-way repeated measurements ANOVA with the seven-step repeated measurements factor time point post glutamate treatment (0, 10, 20, 30, 40, 50, 60 min) and the two-step factor PTEN genotype (PTEN-L:EGFP, PTEN:EGFP). Holm-Sidak’s post hoc tests were performed to compare nuclear mean intensity between PTEN genotypes and to compare nuclear mean intensity between time points within each PTEN genotype.

In Fig. 4d, PLA-complexes indicative of proximities of the secondary antibodies lower than 40 nm were counted in 4 independent wells/condition and compared between glutamate- and PBS treated cells by two-sided, unpaired t-test with Sidak-Holm’s correction of multiple comparisons. Alpha error was set to 0.05. P-values of < 0.05 were considered significant.

All graphics were created with GraphPad (Version 6.07). In Fig. 2b, data which were derived from three independent experiments, were shown as means with standard deviations. In Fig. 3b, n = 20–30 cells per genotype were analysed and data were presented as means with 95% confidence intervals. In Figs 3c and 4d data were shown as interquartile ranges (the box represents first quartile, median and third quartile) with each dot signifying an independent data point and whiskers signifying minimum and maximum.

### Methods to prevent bias

Wells on cell culture plates were randomly assigned to treatment with lentiviral particles delivering different PTEN variants. In experiments in which parallel control plates were used, plates were randomly assigned to control or experimental condition (OGD or washing control). Image analysis was performed blinded to the PTEN variant expressed.

## Supplementary information

Supplementary information

Cellular distribution of PTEN-L:EGFP after glutamate stress

Cellular distribution of PTEN:EGFP after glutamate stress

Cellular distribution of PTEN-L NLS:EGFP after glutamate stress

Supplementary Table 1

Supplementary Table 2

## Data Availability

The cell microscopy videos, images and western blot datasets generated and analysed during the current study are available in the figshare repository 10.6084/m9.figshare.c.4339814^46^. All data sets and detailed protocols are available upon request to the corresponding author.
