# Supplementary material for: Unique properties of PTEN-L contribute to neuroprotection in response to ischemic-like stress
Source: Sci Rep. 2019 Feb 28;9:3183. doi: 10.1038/s41598-019-39438-1 (PMC6395706; doi:10.1038/s41598-019-39438-1)
Supplement: Supplementary file 1 — Supplementary information [file 41598_2019_39438_MOESM1_ESM.pdf]

SUPPLEMENTARY INFORMATION TO:

UNIQUE PROPERTIES OF PTEN-L CONTRIBUTE TO NEUROPROTECTION IN RESPONSE TO ISCHEMIC-LIKE STRESS

Magdalena C. E. Jochner[1,2,6]
Junfeng An[1,2,3]
Gisela Lättig-Tünnemann[1,2]
Marieluise Kirchner[4,5]
Alina Dagane[4]
Gunnar Dittmar[4,9]
Ulrich Dirnagl[1,2,6,7]
Britta J. Eickholt[8]
Christoph Harms[1,2,6*]

Author affiliations

[1]Charité – Universitätsmedizin Berlin, corporate member of Freie Universität Berlin, Humboldt Universität zu Berlin, and Berlin Institute of Health, Neurocure Cluster of Excellence, Department of Experimental Neurology, Germany
[2]Center for Stroke Research Berlin, Charité – Universitätsmedizin Berlin, Germany
[3]Medical Research Centre, The First Affiliated Hospital of Zhengzhou University, Zhengzhou, China
[4]Max Delbrück Centre for Molecular Medicine (MDC), Proteomics Platform, Robert-Rössle-Straße 10, 13125, Berlin, Germany
[5]Berlin Institute of Health (BIH), 10178 Berlin, Germany, Proteomics Platform, Germany
[6]Berlin Institute of Health (BIH), 10178 Berlin, Germany, QUEST – Centre for Transforming Biomedical Research, Germany
[7]German Centre for Neurodegenerative Diseases (DZNE), Berlin, Germany
[8]Charité – Universitätsmedizin Berlin, Institute of Biochemistry, Germany
[9]Proteome and Genome Research Laboratory, Luxembourg institute of Health, 1a Rue Thomas Edison, 1224 Strassen, Luxembourg

*Correspondence and requests of materials should be addressed to C.H. (email: christoph.harms@charite.de)

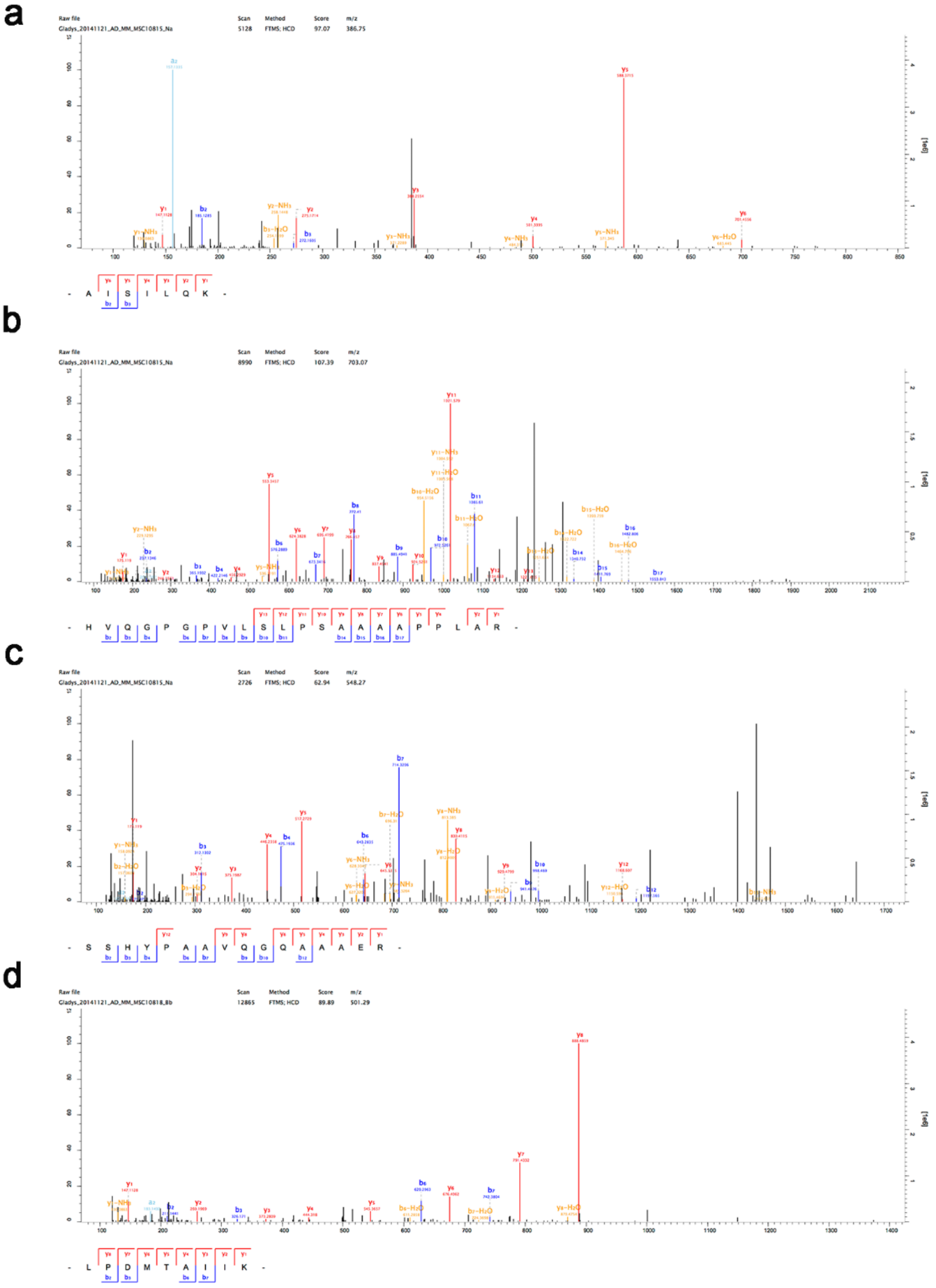

Supplementary Figure 1 | Spectra of four tryptic peptides matched to the PTEN-L sequence. Mouse brain lysates and primary neuronal cell culture lysates were used to identify the PTEN-L isoform by proteomics analysis. (**a-d**) show MS/MS spectra of four tryptic peptides, located in the N-terminus of the PTEN-L form as obtained by LC-ESI-MS analysis. The identity of the peptides was verified by the presence of complementary b- (N-terminal fragment ions) and y-ions (C-terminal fragment ions).

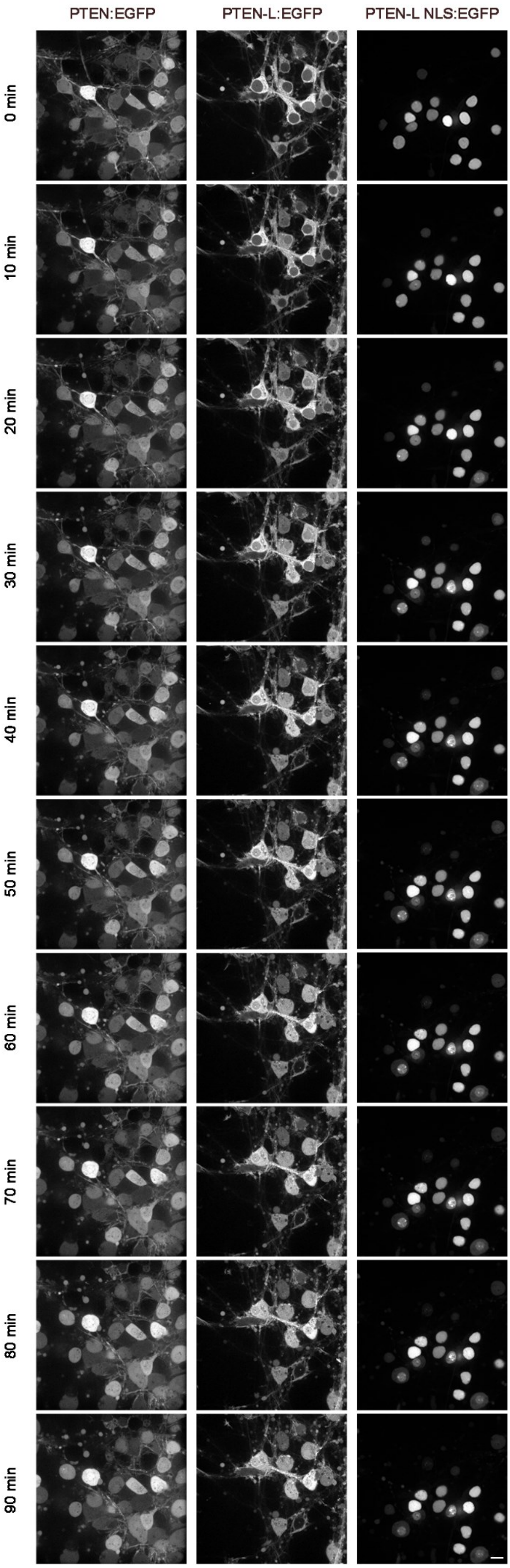

Supplementary Figure 2 | Representation of time lapse movies, which were recorded after neurons expressing different PTEN variants were exposed to 50 µM glutamate. Primary neurons derived from conditional PTEN knockout mice were replaced with different PTEN variants. At DIV 9 neurons were stressed with 50 µM glutamate and pictures were taken every 10 minutes for 90 minutes in a spinning disc confocal microscope.

Representative images of each genetic condition over time are displayed here. PTEN:EGFP was evenly distributed in the nucleus and cytosol of the cells. PTEN-L:EGFP localized mostly in the cytosol and translocated to the nucleus in response to glutamate stress. PTEN-L NLS:EGFP localized exclusively in the nucleus of neurons.

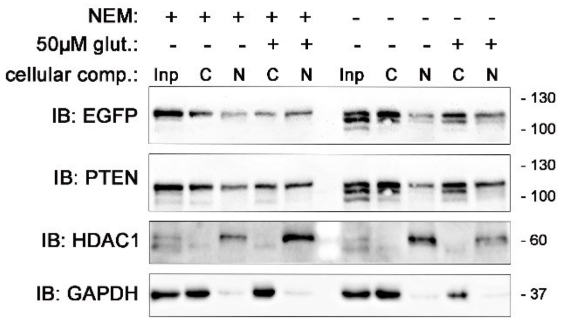

Supplementary Figure 3 | Cell fractionation of PTEN-L:EGFP expressing neurons treated with 50µM glutamate or PBS. Primary neurons replaced with PTEN-L:EGFP were harvested 60 min after treatment with 50µM glutamate or PBS at DIV 9. Half of the samples were harvested with buffers containing an inhibitor of de-ubiquitinating and de-sumoylating enzymes, 20 mM N-Ethylmaleimide (NEM). Nuclear and cytosolic fractions were separated and immunoblotted with antibodies against EGFP, PTEN, HDAC1 and GAPDH. An increase of nuclear PTEN-L:EGFP is visible in glutamate treated neurons irrespective of NEM. PTEN- and EGFP-positive double bands at 110 kD appear in neuronal lysates harvested without NEM, but only the upper band remains when NEM was added to harvesting buffers.

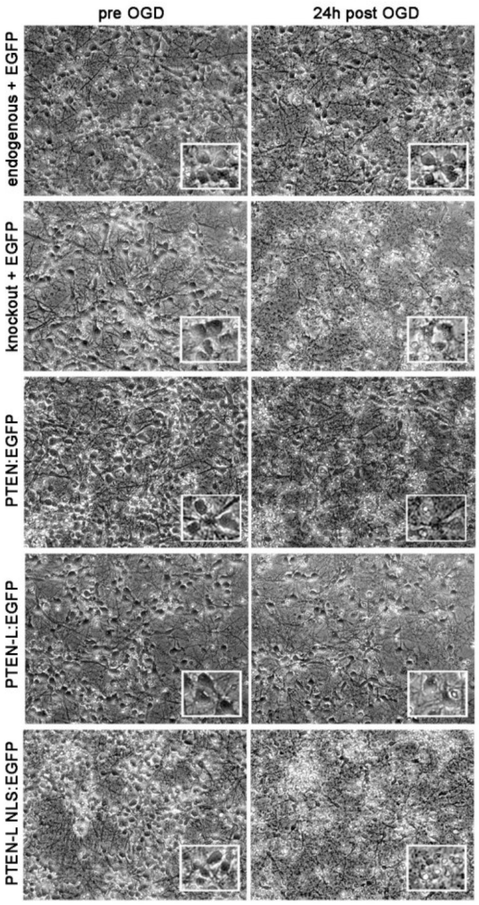

Supplementary Figure 4 | Representative images of neurons expressing different PTEN variants before and 24 h after Oxygen-Glucose deprivation (OGD). Primary neuronal cultures derived from conditional PTEN knockout mice were replaced with different PTEN variants and subjected to OGD at day in vitro 9. Pictures were taken before and 24 h after OGD to control for cell death. Representative images show less cell death in PTEN-L:EGFP replaced neurons compared to PTEN knockout neurons and cultures expressing PTEN-L NLS:EGFP or PTEN:EGFP.

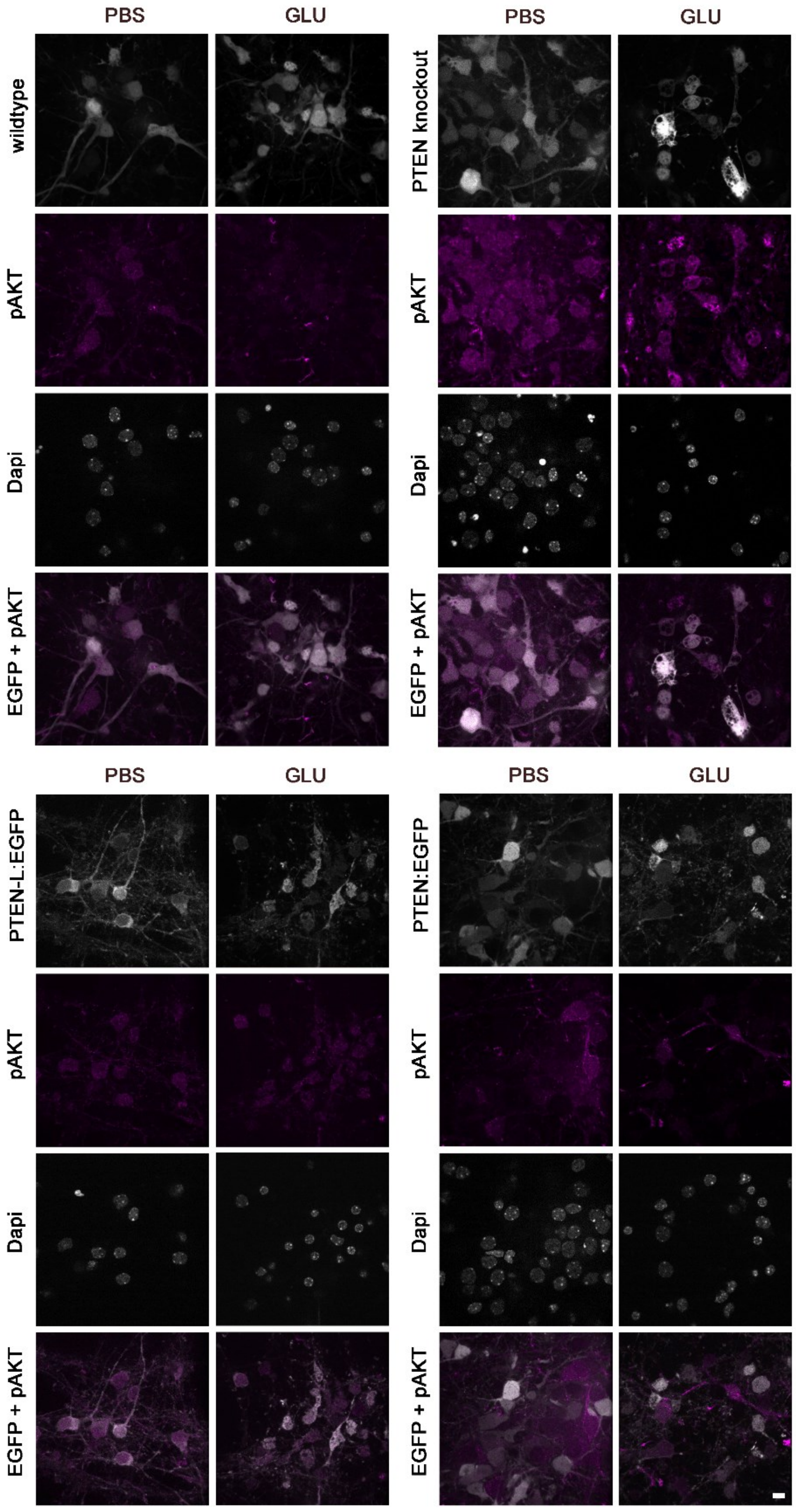

Supplementary Figure 5 | Immunocytochemistry against pAKT in neurons expressing different PTEN variants after 50 µM glutamate or PBS treatment. Primary neurons replaced with PTEN-L:EGFP or PTEN:EGFP as well as PTEN knockout and wildtype neurons were treated with 50µM glutamate or PBS and fixed for immunocytochemistry after 3 hours. A primary antibody against phosphorylated AKT was applied, followed by an Alexa 647 secondary antibody. Images show hypertrophic cell somas and an overall increase in AKT phosphorylation in PTEN knockout neurons. In response to glutamate treatment, no visible change in AKT phosphorylation was observed in any genetic condition. The scale bar represents 10 µM.

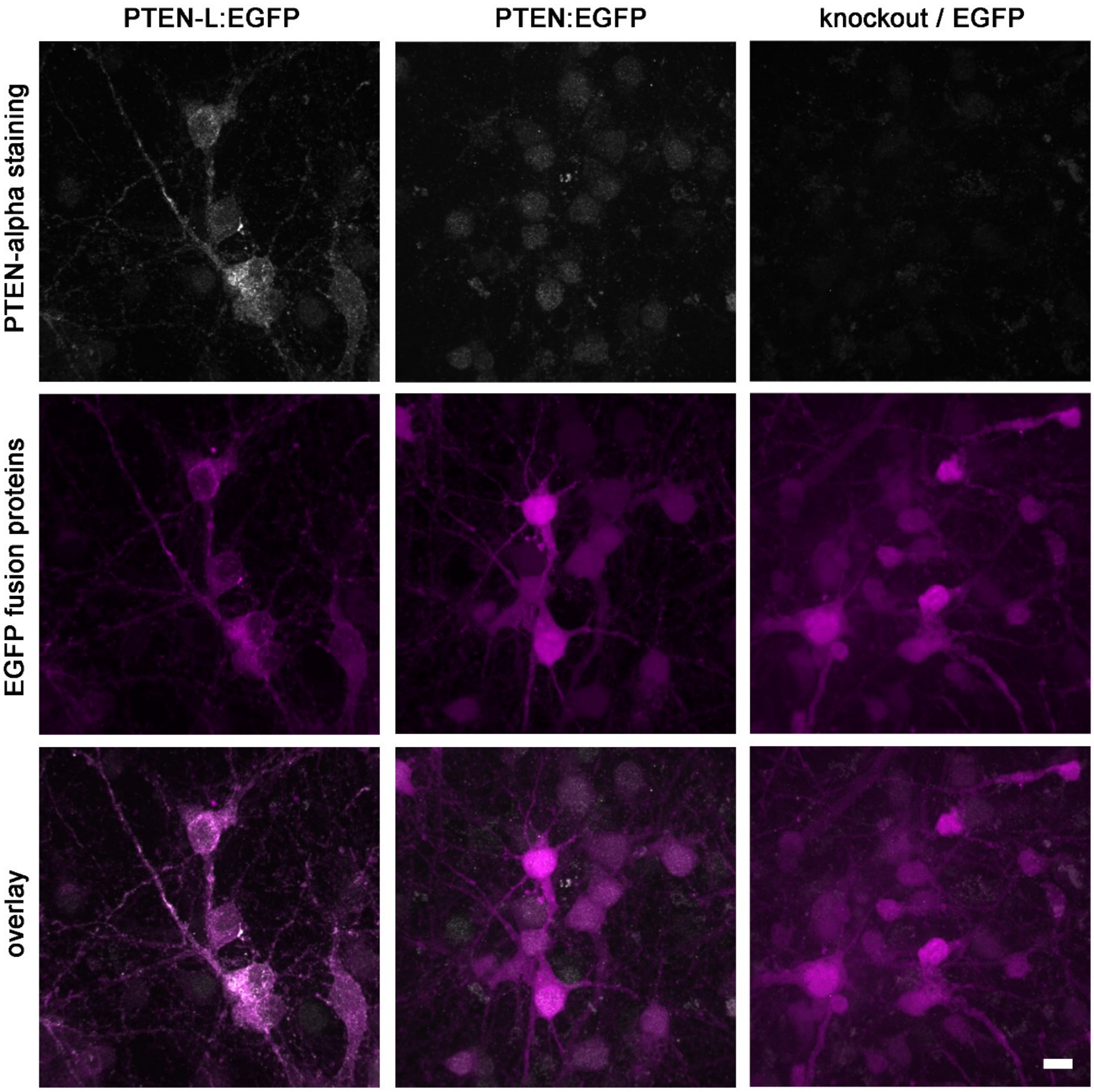

Supplementary Figure 6 | Test of PTEN-alpha antibody. It was tested if the PTEN-alpha antibody specifically detects PTEN-L via immunocytochemistry. When applied in 1:1000 dilution to neurons replaced with PTEN-L:EGFP, the signals of the Alexa 647 secondary antibody and the EGFP fusion protein overlapped. No such overlap was observed in neurons replaced with PTEN:EGFP or PTEN knockout neurons.

Supplementary Table 1 | Cytosolic PTEN and PTEN-L binder at baseline and after 60 min of glutamate treatment (significance cut-off: p-value ≤ 0.05, log_2 t-test difference > 2).

Supplementary Table 2 | Nuclear PTEN and PTEN-L binder at baseline and after 60 min of glutamate treatment (significance cut-off: p-value ≤ 0.05, log_2 t-test difference > 2).
